# Prolonged Hemiplegic Migraine Led to Persistent Hyperperfusion and Cortical Necrosis: Case Report and Literature Review

**DOI:** 10.3389/fneur.2021.748034

**Published:** 2021-10-27

**Authors:** Yacen Hu, Zhiqin Wang, Lin Zhou, Qiying Sun

**Affiliations:** ^1^Department of Geriatric Neurology, Xiangya Hospital, Central South University, Changsha, China; ^2^National Clinical Research Center for Geriatric Disorders, Xiangya Hospital, Central South University, Changsha, China

**Keywords:** hemiplegic migraine, irreversible neurological deficit, cortical necrosis, hyperperfusion, patent foramen ovale (PFO)

## Abstract

Hemiplegic migraine (HM) is a rare subtype of migraine characterized by aura of motor weakness accompanied by visual, sensory, and/or speech symptoms. Aura symptoms usually resolve completely; permanent attack-related deficit and radiographic change were rare. Here, we reported a case presented with progressively aggravated hemiplegic migraine episodes refractory to medication. He experienced two prolonged hemiplegic migraine attacks that led to irreversible visual impairment and cortical necrosis on brain MRI. Multimodal MRI during attack showed persistent vasodilation and hyperperfusion in the affected hemisphere associated with deterioration of clinical symptoms and worsening of brain edema. Patent foramen ovale (PFO) was found on the patient. PFO closure resulted in a significant reduction of HM attacks. This case indicated that prolonged hemiplegic migraine attack could result in irreversible neurological deficit with radiographic changes manifested as cortical necrosis. Persistent hyperperfusion might be an important factor contributing to prolonged attack and persistent attack-related neurological deficit. We recommend screening for PFO in patients with prolonged or intractable hemiplegic migraine, for that closure of PFO might alleviate the attacks thus preventing the patient from disabling sequelae.

## Introduction

Hemiplegic migraine (HM) is characterized by aura of motor weakness accompanied by visual, sensory, and/or speech symptoms followed by migraine headache (1). According to the International Classification of Headache Disorders, 3rd edition (ICHD-3) criteria, aura of HM should be fully reversible (2). Although little is known about imaging manifestations during HM attack, swelling and/or cortical hyperintensity on T2/FLAIR-weighted MRI images have been described, which were mostly reversible after attack resolution (1).

Studies have suggested a possible association between patent foramen ovale (PFO) and migraine with auras (3). Percutaneous PFO closure has been reported to reduce the burden of migraineurs with PFO (4). Report of PFO closure in patients with HM is rare.

## Case Presentation

**Figure 1:**
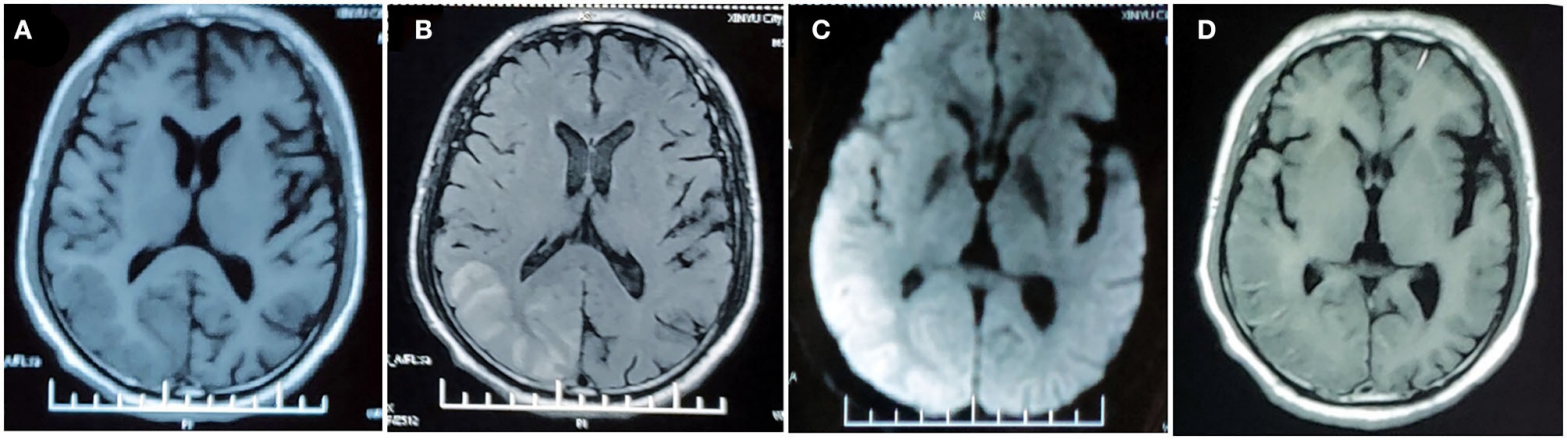
MRI findings in the first prolonged attack. MRI performed 5 days after attack onset demonstrates right temporo-occipital lobe hypointensity on T1-weighted image **(A)** and hyperintensity on T2-FLAIR image **(B)** with restricted diffusion on DWI **(C)**. MRI performed 4 weeks after symptom onset demonstrated right temporal–occipital cortical hyperintensity on T1-weighted images **(D)**.

A 53-year-old man was admitted to our hospital because of severe left-sided headache for 1 week. He reported a long history of migraine with aura since childhood. The migraine was always preceded by aura of hemiparesis and hemianesthesia. Severe pulsating headache started 20 min after aura, lasting for hours with photophobia and vomiting. Aura completely resolved 30 min after headache onset. The attacks were usually triggered by colds, fatigue, insufficient sleep, or mental stress with a frequency of approximately one episode per year. Since age 20, the episode aggravated with increased frequency (7–8 attacks per year), prolonged headache (24~48 h), additional visual aura symptom, and prolonged aura duration (hours, sometimes days after headache resolution). At the age of 51, he suffered the first severe prolonged attack. After continuous heavy labor work for hours, he developed weakness and numbness progressively spreading from his left hand to left arm, face, and leg over the course of 20 min. This was followed by throbbing right-sided headache and visual symptoms, evolving from scintillating scotoma to left homonymous hemianopia within 24 h. Brain MRI at 5 days after attack onset showed right temporal–occipital cortical edema with restricted diffusion. Symptoms lasted for 3 weeks with incomplete recovery of visual impairment, leaving a left visual field defect. Repeated MRI performed 4 weeks after symptom onset showed hyperintensity on T1-weighted image in right occipital–temporal cortex, suggestive of cortical necrosis (Figure 1).

One week before admission to our hospital, he experienced the second prolonged attack after sleep deprivation for overnight work. Weakness and numbness started in the right hand and rapidly extended to the homolateral side of the face and leg, accompanied by speech disfluency and scintillating scotoma in right hemifield, followed by a severe pulsating headache within 10 min. Neurological symptoms deteriorated with aphasia, confusion, and right homonymous hemianopia the next day. Neurological symptoms deteriorated with aphasia, confusion, and right homonymous hemianopia the next day. Headache was refractory to ibuprofen and tramadol; the neurological symptoms persisted without resolution.

**Figure 2:**
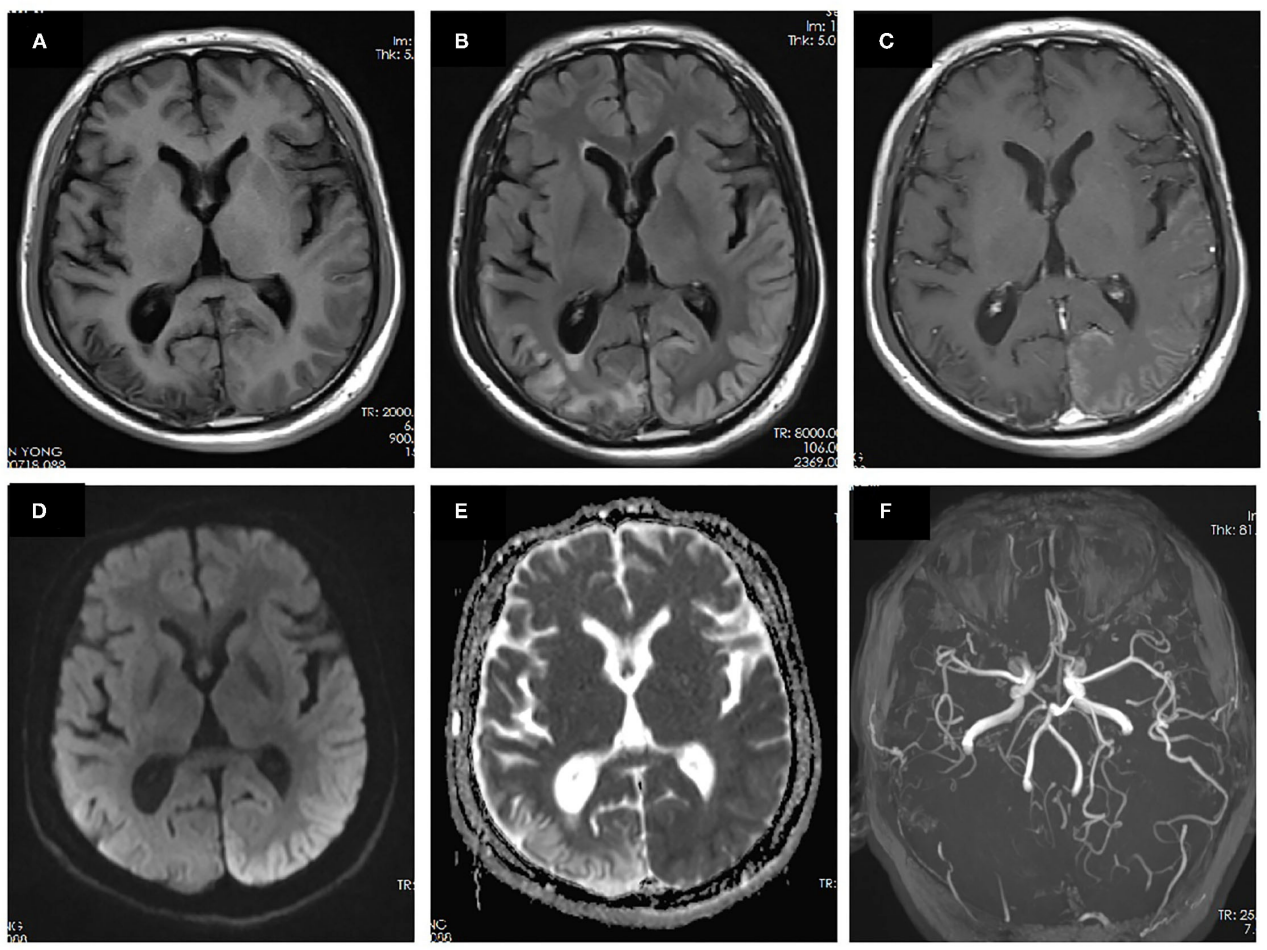
MRI performed 10 days after onset of the second prolonged attack. Right temporal-occipital hyperintensity on T1-weighted images **(A)** with gliosis on T2-FLAIR images **(B)** suggests the chronic stage of cortical necrosis caused by first prolonged attack. Left temporal–occipital cortex hyperintensity on T2-FLAIR images **(B)** with gyriform enhancement **(C)**, restricted diffusion **(D)**, and normal apparent diffusion coefficient (ADC) **(E)** suggests the subacute stage of cortical necrosis caused by second prolonged attack. MRA demonstrated vasodilation of the branches of the left middle cerebral artery (MCA) and posterior cerebral artery (PCA) **(F)**.

On admission, the patient was afebrile with normal vital signs. Neurological examination showed mild decreased alertness, right-sided paresis and paresthesia, mixed aphasia, slight right gaze palsy, and bilateral visual field defect predominately in the right side. Laboratory testing including blood count, electrolytes, lactate acid, creatine kinase, coagulation panel, rheumatoid factor, antinuclear antibodies (ANA), antinuclear cytoplasmic antibodies (ANCA), lupus anticoagulant, antiphospholipid antibodies, and autoimmune encephalitis panel were normal. Lumbar puncture showed normal intracranial pressure. Cerebrospinal fluid (CSF) examination revealed normal cell count, glucose content, protein, and lactate acid level. CSF tests for infectious disease including gram stain, bacterial cultures, treponema pallidum hemagglutination assay, cryptococcus antigen, and the next-generation sequencing for pathogens were negative. Consecutive electroencephalogram (EEG) during attack showed slow background activity over the left hemisphere without epileptic discharge. Brain MRI performed 10 days after the episode onset showed hyperintensity of the right temporal–parietal–occipital cortex on T2-FLAIR images with gyriform enhancement and diffusion restriction. MRA demonstrated vasodilation of the branches of the left middle cerebral artery (MCA) and posterior cerebral artery (PCA) (Figure 2). Transthoracic echocardiography revealed the presence of PFO. Mitochondrial DNA and whole exon sequence of nuclear DNA were analyzed by targeted region capture and high-throughput sequencing, but failed to detect any significant variant.

**Figure 3:**
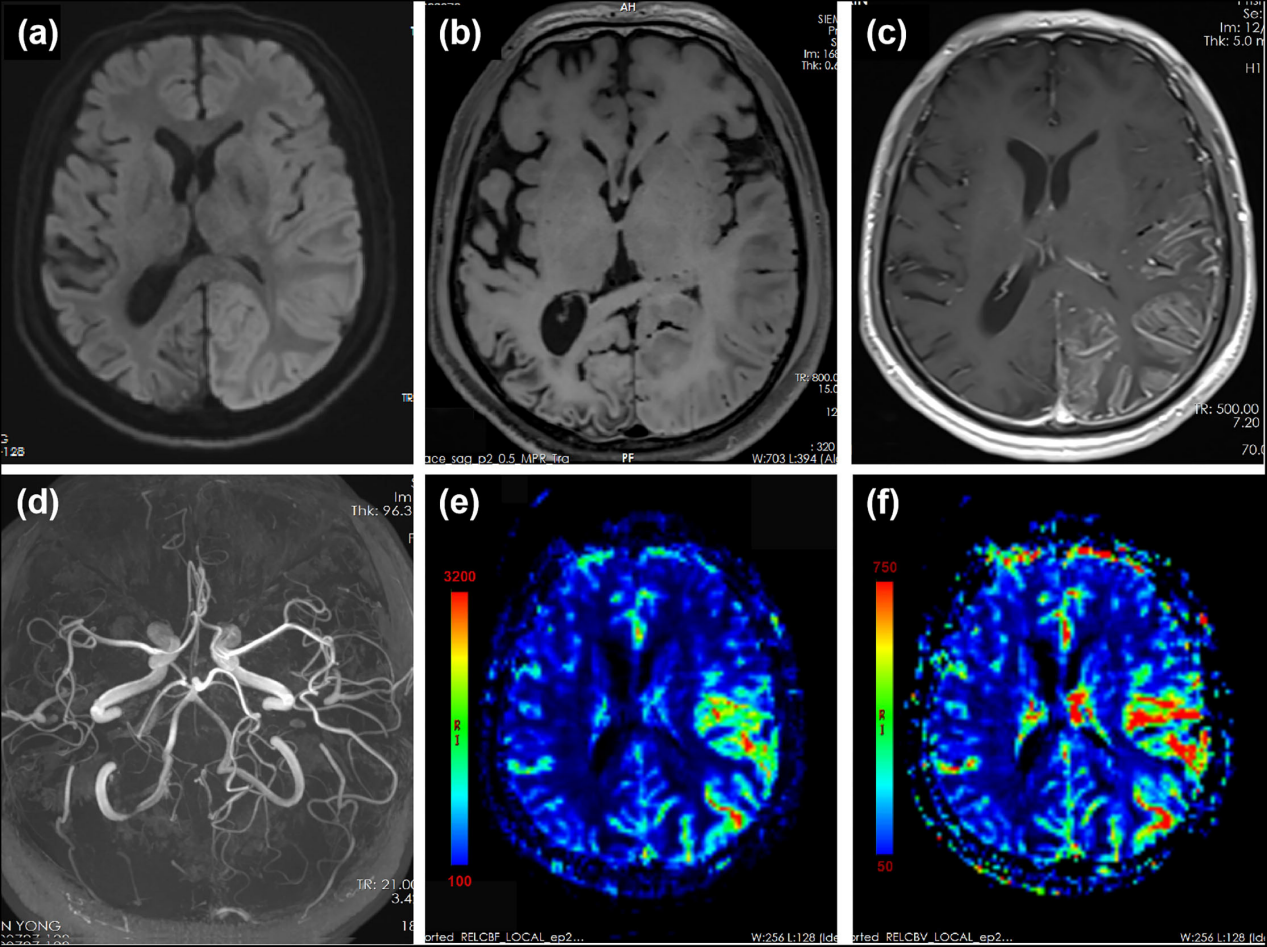
MRI performed 15 days after the onset of the second prolonged attack. DWI showed extensive cortical edema with diffusion restriction involving the left hemisphere **(a)**. T1-weighted images showed slight hyperintensity in left temporal–occipital lobe and volume loss of right temporal–occipital lobe, suggestive of subacute and chronic stage of cortical necrosis, respectively **(b)**. Gadolinium-enhanced T1-weighted images showed cortical enhancement in the left temporal–occipital lobe **(c)**. MRA showed dilation of left MCA and PCA **(d)**. PWI showed increased cerebral blood flow (CBF) **(e)** and cerebral blood volume (CBV) **(f)** in the left hemisphere. Region of interest (ROI) placed on the left temporal–parietal area showed a mean relative CBF value of 486.1 compared with that of 123.1 on the right temporal–parietal area. ROI placed on the left temporal–parietal area showed a mean relative CBV value of 533.1 compared with that of 129.8 on the right temporal–parietal area.

The patient had no risk factors for ischemia stroke. None of his parents or siblings experienced migraine headaches or auras. Given the negative family history and molecular genetic analysis, as no other etiology could be evidenced, the diagnosis of prolonged sporadic hemiplegic migraine was considered the most likely. The patient was treated with diclofenac (75 mg, qd) to relieve headache, flunarizine (10 mg, qn) and topiramate (50 mg, bid) for migraine prophylaxis, and glycerine fructose to alleviate cerebral edema. The headache relieved slightly while neurological impairment symptoms persisted without resolution. On day 15 since the second episode onset, headache deteriorated with aggravated limb weakness and worsened consciousness. Repeated brain MRI revealed aggravated cortical edema involving the entire left hemisphere. MRA showed prominent vasodilation of left MCA and PCA while PWI showed hyperperfusion in corresponding region (Figure 3). Headache and neurological symptoms subsided gradually and spontaneously during the next 2 weeks and the patient was discharged on prophylactic dose of flunarizine (10 mg, qn) and topiramate (50 mg, bid).

During the following 2 months, HM episode recurred four times even though the patient adhered to the prophylaxis. Two months later, the patient was hospitalized again and a percutaneous procedure of PFO closure was performed successfully. The patient experienced substantially reduced frequency of episodes after the PFO closure, without taking any prophylactic medication for migraine. On 18-month follow-up, he reported only two episodes of mild headache with mild weakness and numbness lasting for <3 h. However, visual impairment did not resolve completely in bilateral visual field.

## Discussion

Aura symptoms of HM usually fully resolve within 24 h. Although cases with slow recovery and neurological symptoms that lasted up to weeks and months have been described, permanent attack-related deficit was rare. Several case reports showed diffusive or hemispheric cortical edema with diffusion restriction during acute phase of HM attacks, which were mostly reversible after attack resolution. To our knowledge, only two cases reported attack-related permanent sequelae with corresponding irreversible neuroimaging changes in hemiplegic migraine (5, 6). Our patients, who had a long history of recurrent attacks of HM, developed irreversible visual deficits as a result of two prolonged attacks that were associated with neuroimaging changes manifested as cortical necrosis.

Cortical necrosis is a permanent injury characterized by selective necrosis of the cerebral cortex. It is generally caused by severe long-lasting cerebral metabolism impairment and energy depletion. Image features are characterized by gyriform hyperintensity of the cerebral cortex on FLAIR and gadolinium-enhanced images in acute phase. Hyperintensity on T1-weighted images often appears 2 weeks after the events and may last up to 2 years (7). In our patient, the gyriform enhancement and hyperintensity on FLAIR and T1-weighted images were consistent with the characteristics of cortical necrosis.

Cortical necrosis has been reported in both mitochondrial encephalopathy as well as convulsive and non-convulsive epilepsy status, which should be considered as differential diagnosis for this patient. Mitochondrial encephalopathy was not considered based on normal serum lactate level, absence of other clinical features, and negative genetic tests. Epilepsy and migraine are common comorbidities and sometimes difficult to be clinically differentiated. They share similar trigger factors, course of attacks, and accompanying neurological deficit including changes of consciousness. They even overlap on pathophysiological and genetic mechanisms. Different mutations in causative genes of familial hemiplegic migraine can produce either migraine, epilepsy, or both. In this case, epilepsy cannot be completely ruled out, although it was considered less likely as the cause leading to cortical necrosis for the absence of typical symptoms/signs suggestive of epilepsy, and lack of epileptic-like discharge on repeated long-term EEG.

Cortical necrosis has also been reported in migrainous infarction (8), an uncommon complication of migraine with aura, defined as cerebral ischemic infarction that occurs during the attack of migraine with aura. In our case, migrainous infarction should be considered. First, coexistence of common risk factors for ischemic infarction, including hypertension, diabetes, tobacco use, and hypercoagulability, was ruled out. Second, cortical lesion extending over two blood vessel territories argue against embolic infarction caused by PFO or macrovascular etiologies. On the other hand, deterioration of neurological symptoms and aggravation of brain edema were associated with persistent vasodilation and hyperperfusion in the affected hemisphere during the second prolonged attack, indicating that ischemia is probably not the only factor leading to prolonged symptoms and cerebral edema.

In classic migraine with aura, it is believed that the aura symptoms are associated with the initial focal hypoperfusion whereas headaches are associated with later vasodilation. The findings observed in our patient that aura symptoms are persistent when imaging studies demonstrate vasodilation and hyperperfusion seem opposite this theory, suggesting that persistent neurological deficit in HM may be caused by different mechanisms. In fact, both hypo- and hyper-perfusion in the affected hemisphere during HM attacks have been documented (9, 10). Several cases showed biphasic changes—initial hypoperfusion followed by persistent hyperperfusion—during prolonged aura (11), although the exact alternating time was unclear, ranging from 5.5 to 18 h after the attack onset. As summarized in Table 1, vasodilation or hyperperfusion ipsilateral to affected hemisphere was observed in most prolonged attack with persistent neurological deficit more than 1 week, especially when perfusion was measured in the later stage of acute phase. In those cases, the time window of detecting hyperperfusion in affected hemisphere varied from 6 h to 16 days after attack onset. Only one case reported persistent neurological deficit with hypoperfusion during attack, in which the cerebral perfusion was evaluated at very early phase within 4.5 h after attack onset, suggesting that the reversal from hypoperfusion to hyperperfusion may be earlier than anticipated if biphasic pattern is a universal phenomenon during HM attack. Most patients including the present case were not evaluated for cerebral perfusion during the early stage, thus might have missed the initial hypoperfusion stage. As a result, continuous cerebral perfusion monitoring covering early and late stage during attack is valuable to clarify the biphasic pattern of cerebral perfusion and the exact reversal timing.

**Table 1:** Persistent neurological deficit with perfusion studies in HM attacks.

**Reference**	**Age^a^**	**Sex^b^**	**Neurological deficit^c^**	**Attack** **duration^d^**	**Neuroimaging**		**Prognosis^g^**
					**Time^e^**	**Neuroimaging findings^f^**	
(13)	13/10.5	F	Hemiplegia (R), seizure, aphasia	2 w	During attack	MRI: hemispheric swelling (L) MRA: MCA (L) dilation	Right hemiplegia (>2 w)
(14)	44/34	M	Hemiplegia (R), coma, fever, dysphasia, seizure	1.5 m	6 d	SPECT: normal perfusion	Unconsciousness, bedridden (8 m)
					1 m	MRI: hemispheric edema (L)	
(15)	21/nd	M	Hemiplegia (R), aphasia	10 d	<6 h	DWI and MRA: normal PWI: hemispheric hyperperfusion (L)	Aphasia (>2 w)
					4 d	PWI: frontal lobe hyperperfusion (L)	
(9)	35/12	F	Hemiplegia (R), confusion, visual defect, aphasia	10 d	4 d	MRA: MCA (L) dilation SPECT: hyperperfusion (L)	ND
(16)	33/10	F	Hemiplegia (L), visual defect, disorientation, confusion	>1 w	1 w	MRI: hemispheric cortical swelling with diffusion restriction and enhancement (R) PWI: temporoparietal hyperperfusion (R)	Complete recovery (3 m)
(5)	50/40	M	Hemiplegia (A), aphasia, confusion	>2 w	2 w	MRI: diffusive cortical edema with enhancement MRA: MCA and PCA dilation ipsilateral to cortical lesions FDG–PET: increased FDG uptake within cortical lesions	Unable to live alone (4 m)
(17)	20/8	F	Hemiplegia (L), seizure	>11 d	1 d	MRI: hemisphere swelling (R) with dural enhancement MRA: MCA and PCA dilation (R) PWI: hemispheric hyperemia (R)	Left arm weakness (2 w)
					6 d	CTA: persistent MCA and PCA dilation (R) CTP: hemispheric hyperperfusion (R)	
(18)	64/40	M	Hemianopsia, cognitive impairment	>16 d	5 d	MRI: temporal (R) and occipital (R) cortical edema with diffusion restriction and enhancement MRA: MCA and PCA dilation (R) PWI: temporal (R) and occipital (R) hyperperfusion	Visual field defect (50 d)
					9 d	PWI: temporal (R) and occipital (R) hyperperfusion	
					16 d	Xe_CT: temporal (R) and occipital (R) hyperperfusion	
(19)	21/14	M	Hemiplegia (R), hemianopia (R), aphasia	2 w	8 h	DWI and PWI: normal	Aphasia (2 w)
					7 d	PET: hemispheric hyperperfusion (L)	
(20)	22/15	F	Hemiplegia (R), hemianesthesia, photophobia, aphasia, cognitive dysfunction	6 w	<24 h	MRI: normal	Cognitive deficits and hemianesthesia (9 m)
					10 d	MRI: hemispheric cortical edema with diffusion constriction (L)	
(21)	27/8	M	Hemiplegia (R), visual symptoms, hemianesthesia, cognitive slow, confusion	1 w	During attack	DWI: normal ASL: occipital, parietal, and posterior temporal lobe hyperperfusion (L)	Speech influency (6 m)
(10)	44/39	M	Hemiplegia (L), fever, seizure, photophobia	9 d	4.5 h	MRI: normal PWI: hypoperfusion	Cognitive impairment (4 m)
(22)	43/nd	M	Hemiplegia (L), confusion	>1 w	5 d	MRI: hemispheric cortical edema with enhancement (R) PWI: hyperperfusion	ND
(23)	7/nd	M	Hemiplegia (L), fever, confusion	5 w	1 w	DWI: hemispheric edema (R) MRA: hemispheric vasodilation (R) ASL:hemispheric hypererfusion (R)	Hemiplegia (>5 w)

a *Age at attack/age at HM onset; nd, not described*.

b *M, male; F, female*.

c *R, right; L, left; A, alternating*.

d *h, hour; d, day; w, week; m, month*.

e *Time after attack onset*.

f *DWI, diffusion-weighted imaging; MRA, magnetic resonance angiography; PWI, perfusion-weighted imaging; ASL, arterial spin labeling MRI; Xe_CT, xenon_CT; SPECT, single photon emission computed tomography; FDG, fluorodeoxyglucose; PET, positron emission tomography; MCA, middle cerebral artery; PCA, posterior cerebral artery*.

g *Residual symptoms (persistent duration)*.

The mechanism underlying vasodilation and hyperperfusion remains unknown. Hypothesis suggested that vasodilation was caused by increased energy demand due to intrinsic neuron activation during prolonged HM attack. When cerebral perfusion is not sufficient, energy depletion and metabolite accumulation occur, leading to hypermetabolic neural necrosis. Another hypothesis speculated that vasodilation is caused by impaired cerebral artery autoregulation. Persistent vasodilatation could lead to hyperperfusion brain damage, which could result in brain edema and cortical necrosis on MRI (12). Also, given the biphasic neurovascular changes observed in prolonged HM attacks, it is speculated that persistent vasodilation or hyperperfusion might be a compensatory phenomenon secondary to extremely severe initial hypoperfusion. Based on the aforementioned hypotheses, persistent hyperperfusion might indicate serious cerebral metabolic disturbance, which may result in irreversible neurological impairment. In our patient, persistent hyperperfusion lasting up to 15 days after attack onset ultimately leads to cortical necrosis and permanent neurological deficit.

Another notable aspect in our case was the presence of a PFO. Observational studies suggested that PFO closure may reduce migraine attacks (4), but results from randomized trials were controversial (24, 25). Due to the rarity of HM, only three cases reported the closure of PFO in HM patients. All of the three patients gained partial or complete relief from attacks after PFO closure (26–28). In our case, disabling attacks refractory to medication were remarkably reduced after PFO closure without taking prophylactic medication, adding to the evidence that HM patients with PFO might benefit from PFO closure. However, in the natural history of HM, gradual relief at adulthood or long interval without attacks could occur unpredictably, which we could not completely rule out in our case. However, given the previous history that frequency and severity of attacks gradually increase with age, sudden spontaneous remission in our patients is less unlikely. In addition, we noticed that migraine attacks may not cease completely after PFO closure in our case and previous reported cases, suggesting that PFO was not the only factor contributing to HM attacks.

## Conclusion

This case indicates that prolonged hemiplegic migraine attack, although rare, could result in irreversible neurological deficit with neuroimaging changes manifested as cortical necrosis. Prolonged hyperperfusion in the affected hemisphere may indicate prolonged attack and even irreversible brain damage. Continuous cerebral perfusion monitoring is valuable to clarify the neurovascular changes during HM attacks, and to understand the mechanisms underlying persistent neurological deficit. Closure of PFO might alleviate the attacks, thus preventing the patient from disabling sequelae. The association between PFO and HM, and the efficacy of PFO closure in HM need to be confirmed in randomized controlled studies. However, for patients with prolonged or intractable hemiplegic migraine, screening for PFO is recommended as patients might benefit from the closure of PFO.

## Data Availability Statement

The raw data supporting the conclusions of this article will be made available by the authors, without undue reservation.

## Ethics Statement

Written informed consent was obtained from the individuals' legal guardian/next of kin for the publication of any potentially identifiable images or data included in this article.

## Author Contributions

YH and ZW collected the clinical data, reviewed the literature, and drafted the article. QS and LZ designed the study, oversaw data acquisition, supervised the initial drafting, and critically revised the article. All authors read and approved the final article.

## Funding

This work was supported by grants from the National Natural Science Foundation of China (81401060, 82171256) and Natural Science Foundation of Hunan Province, China (2020JJ5927, S2021JJMSXM1486).

## Conflict of Interest

The authors declare that the research was conducted in the absence of any commercial or financial relationships that could be construed as a potential conflict of interest.

## Publisher's Note

All claims expressed in this article are solely those of the authors and do not necessarily represent those of their affiliated organizations, or those of the publisher, the editors and the reviewers. Any product that may be evaluated in this article, or claim that may be made by its manufacturer, is not guaranteed or endorsed by the publisher.

## References

[1] Di Stefano V Rispoli MG Pellegrino N Graziosi A Rotondo E Napoli C. Diagnostic and therapeutic aspects of hemiplegic migraine. J Neurol Neurosurg Psychiatry. (2020) 91:764–71. 10.1136/jnnp-2020-322850 32430436 PMC7361005

[2] Headache Classification Committee of the International Headache Society (IHS). The international classification of headache disorders, 3rd edition. Cephalalgia. (2018) 38:1–211. 10.1177/0333102417738202 29368949

[3] Takagi H Umemoto T ALICE (All-Literature Investigation of Cardiovascular Evidence) Group. A meta-analysis of case-control studies of the association of migraine and patent foramen ovale. J Cardiol. (2016) 67:493–503. 10.1016/j.jjcc.2015.09.016 26527111

[4] Slavin L Tobis JM Rangarajan K Dao C Krivokapich J Liebeskind DS. Five-year experience with percutaneous closure of patent foramen ovale. Am J Cardiol. (2007) 99:1316–20. 10.1016/j.amjcard.2006.12.054 17478165

[5] Cha YH Millett D Kane M Jen J Baloh R. Adult-onset hemiplegic migraine with cortical enhancement and oedema. Cephalalgia. (2007) 27:1166–70. 10.1111/j.1468-2982.2007.01369.x 17645764

[6] Dodick D Roarke M. Familial hemiplegic migraine: permanent attack-related neurologic deficits. Headache. (2007) 47:1210–2. 10.1111/j.1526-4610.2007.00889.x 17883529

[7] Siskas N Lefkopoulos A Ioannidis I Charitandi A Dimitriadis AS. Cortical laminar necrosis in brain infarcts: serial MRI. Neuroradiology. (2003) 45:283–8. 10.1007/s00234-002-0887-7 12743663

[8] Morais R Sobral F Cunha G Brito O Santana I. Advanced MRI study of migrainous infarction presenting as cortical laminar necrosis - case report and literature review. Clin Neurol Neurosurg. (2018) 167:82–5. 10.1016/j.clineuro.2018.02.016 29471286

[9] Iizuka T Sakai F Suzuki K Igarashi H Suzuki N. Implication of augmented vasogenic leakage in the mechanism of persistent aura in sporadic hemiplegic migraine. Cephalalgia. (2006) 26:332–5. 10.1111/j.1468-2982.2005.01025.x 16472342

[10] Blicher JU Tietze A Donahue MJ Smith SA Ostergaard L. Perfusion and pH MRI in familial hemiplegic migraine with prolonged aura. Cephalalgia. (2016) 36:279–83. 10.1177/0333102415586064 25948653

[11] Iizuka T Tominaga N Kaneko J Sato M Akutsu T Hamada J. Biphasic neurovascular changes in prolonged migraine aura in familial hemiplegic migraine type 2. J Neurol Neurosurg Psychiatry. (2015) 86:344–53. 10.1136/jnnp-2014-307731 25411546

[12] Kai Y Hamada J Morioka M Yano S Mizuno T Ushio Y. Postoperative hyperperfusion in a patient with a dural arteriovenous fistula with retrograde leptomeningeal venous drainage: case report. Neurosurgery. (2003) 53:228–32. 10.1227/01.NEU.0000069536.64823.F9 12823895

[13] Meaney JF Williams CE Humphrey PR. Case report: transient unilateral cerebral oedema in hemiplegic migraine: MR imaging and angiography. Clin Radiol. (1996) 51:72–6. 10.1016/S0009-9260(96)80226-1 8549055

[14] Hayashi R Tachikawa H Watanabe R Honda M Katsumata Y. Familial hemiplegic migraine with irreversible brain damage. Intern Med. (1998) 37:166–8. 10.2169/internalmedicine.37.166 9550598

[15] Lindahl AJ Allder S Jefferson D Allder S Moody A Martel A. Prolonged hemiplegic migraine associated with unilateral hyperperfusion on perfusion weighted magnetic resonance imaging. J Neurol Neurosurg Psychiatry. (2002) 73:202–3. 10.1136/jnnp.73.2.202 12122185 PMC1737998

[16] Jacob A Mahavish K Bowden A Smith ET Enevoldson P White RP. Imaging abnormalities in sporadic hemiplegic migraine on conventional MRI, diffusion and perfusion MRI and MRS. Cephalalgia. (2006) 26:1004–9. 10.1111/j.1468-2982.2006.01131.x 16886937

[17] Hsu DA Stafstrom CE Rowley HA Kiff JE Dulli DA. Hemiplegic migraine: hyperperfusion and abortive therapy with intravenous verapamil. Brain Dev. (2008) 30:86–90. 10.1016/j.braindev.2007.05.013 17614229

[18] Arai S Utsunomiya H Arihiro S Arakawa S. Migrainous infarction in an adult: evaluation with serial diffusion-weighted images and cerebral blood flow studies. Radiat Med. (2008) 26:313–7. 10.1007/s11604-008-0226-y 18661217

[19] Freilinger T Peters N Remi J Linn J Hacker M Straube A. A case of Sturge-Weber syndrome with symptomatic hemiplegic migraine: clinical and multimodality imaging data during a prolonged attack. J Neurol Sci. (2009) 287:271–4. 10.1016/j.jns.2009.08.050 19733861

[20] Schwedt TJ Zhou J Dodick DW. Sporadic hemiplegic migraine with permanent neurological deficits. Headache. (2014) 54:163–6. 10.1111/head.12232 24117121 PMC4220590

[21] Moreton FC Santosh C McArthur K Muir KW. Cerebral hyperperfusion on arterial spin labeling MRI during CADASIL migrainous encephalopathy. Neurology. (2015) 85:2177–9. 10.1212/WNL.0000000000002214 26567267

[22] Pellerin A Marois C Mezouar N Mokhtari K Leclercq D Law-Ye B. Neuronal injuries evidenced by transient cortical magnetic resonance enhancement in hemiplegic migraine: a case report. Cephalalgia. (2019) 39:323–5. 10.1177/0333102418794481 30092648

[23] Kornbluh AB Chung MG. Teaching neuroimages: transient cytotoxic edema in a child with a novel ATP1A2 mutation. Neurology. (2020) 95:e1441–e2. 10.1212/WNL.0000000000010103 32641521

[24] Tobis JM Charles A Silberstein SD Sorensen S Maini B Horwitz PA. Percutaneous closure of patent foramen ovale in patients with migraine: the PREMIUM trial. J Am Coll Cardiol. (2017) 70:2766–74. 10.1016/j.jacc.2017.09.1105 29191325

[25] Mattle HP Evers S Hildick-Smith D Becker WJ Baumgartner H Chataway J. Percutaneous closure of patent foramen ovale in migraine with aura, a randomized controlled trial. Eur Heart J. (2016) 37:2029–3. 10.1093/eurheartj/ehw027 26908949

[26] Brighina F Gurgone G Gaglio RM Palermo A Cosentino G Fierro B. Case of atypical sporadic hemiplegic migraine associated with PFO and hypoplasia of vertebro-basilar system. J Headache Pain. (2009) 10:303–6. 10.1007/s10194-009-0125-3 19421707 PMC3451752

[27] Lemka M Pienczk-Reclawowicz K Pilarska E Szmuda M. Cessation of sporadic hemiplegic migraine attacks after patent foramen ovale closure. Dev Med Child Neurol. (2009) 51:923–4. 10.1111/j.1469-8749.2009.03466.x 19758362

[28] Perrotta A Gambardella S Ambrosini A Anastasio MG Albano V Fornai F. A novel ATP1A2 gene variant associated with pure sporadic hemiplegic migraine improved after patent foramen ovale closure: a case report. Front Neurol. (2018) 9:332. 10.3389/fneur.2018.00332 29867740 PMC5966544

